# Analytical volume model for optimized spatial radar bat detection in onshore wind parks

**DOI:** 10.1371/journal.pone.0239911

**Published:** 2020-09-30

**Authors:** Jannes Kreutzfeldt, Carolin Floeter, Thies Lingner, Lukas Schmitz-Beuting, Michael Reich, Veit Dominik Kunz

**Affiliations:** 1 Department of Environmental Technology, Faculty of Life Sciences, University of Applied Sciences Hamburg, Hamburg, Germany; 2 Institute of Environmental Planning, Leibniz University Hannover, Hannover, Germany; 3 Department of Process Engineering, Faculty of Life Sciences, University of Applied Sciences Hamburg, Hamburg, Germany; Oak Ridge National Laboratory, UNITED STATES

## Abstract

To develop mitigation measures for the protection of bats in close proximity to onshore wind turbines, new detection techniques covering large-scale environments and techniques, which are able to track individuals are required. Radar based observations, successfully applied in ornithological studies, offer a promising potential, but come with challenges regarding the comparability of measurements and noise interference (ground clutter) from objects within detection range. This paper presents improvements of a commercially available inexpensive pulse radar for 3D spatial detection of bat-sized objects in onshore wind parks. A new analytical spatial detection volume model is presented incorporating calibrated radar data and landscape parameters such as clutter. Computer simulation programs to process the analytical spatial detection volume model were developed. For model calibration, the minimum signal power of the radar was experimentally determined with the radar cross section (RCS) of an artificial bat (similar to *Nyctalus noctula)*, resulting in a maximum detection range of 800 m and a corresponding RCS of 12.7 cm². Additionally, the spatial volume for radar detection was optimized with a clutter shielding fence (CSF). Adjusting the volume model by incorporating a theoretical model of the CSF, an extension of the detection volume by a factor of 2.5 was achieved, while the total volume of a 105° horizontal angular radar image section yields 0.0105 km³. Extrapolation and comparison with state-of-the-art acoustic bat detection result in a 270 times larger volume, confirming the large-scale detection capabilities of the pulse radar.

## Introduction

The vast growing number of wind parks has significant impact on bat populations. Besides causing bat casualties during migration flights, wind turbines (turbines) are also harmful to resident bats [1–4]. Studies from Germany realized between July and September show an average value of 9.5 deceased bats per turbine and year (minimal 0 to maximal 57.5) [1]. Although still under investigation, direct collision with the turbine rotor blades or the barotrauma are currently seen to be the most lethal [5]. This is in strong contrast with the European law, listing all European bat species, their breeding sites and resting places as being protected. There have been several studies covering the activities of bats at turbines, providing guidelines for monitoring bats [1–4, 6–9]. Although several hypothesizes exist, none of the proposed collision risks and attraction effects could be confirmed so far [10, 11]. Shut down algorithms implemented in the turbine controller are therefore based on assumptions of bats temporally limited activities [7, 9, 12]. In order to develop risk mitigation measures and to better understand bats behavior in wind parks, further studies, especially regarding the 3D movement of bats in wind parks are required [2, 3].

Due to their small size, bats are difficult to locate in larger areas such as the periphery of turbines or within a complete onshore wind park. Current state of the art bat detection is based on acoustic detectors, sampling bats ultrasonic calls [6, 8]. However, acoustic detectors have a huge disadvantage, because of the limited detection range of 15 m to 60 m depending on the emitted frequency of the bat and the utilized detector sensitivity [13]. Recent turbine nacelle heights of more than 150 m and rotor diameters exceeding 100 m make observations of bats even more challenging. Therefore, improved detection methods, that cover a large spatial volume are required for further investigation in the proximity of turbines [4].

Detecting bats with radar is a promising way to overcome the range limitations [14, 15]. Furthermore, radar has the huge advantage of tracking individual objects, which is almost impossible with standard acoustic detectors [16, 17]. Analytic description of electromagnetic radiation has been around for more than a half century [18, 19]. So far, most studies presented where mainly carried out by ornithologists utilizing radar technology for the detection of birds [14, 19–28]. Only a few radar studies have been published investigating more complex shapes such as bats [2, 4, 23, 29, 30]. Biological radar studies of insects also gained little coverage so far [31, 32]. However, a few radar systems optimized for the detection and tracking of birds, such as the MERLIN radar from DeTect, the Robin Radar System or the Swiss Birdradar have been developed [33]. Unfortunately, these radar systems are very expensive. Furthermore, optimized radar systems are mostly designed for object detection in high altitudes (up to several kilometers), often implemented with vertical oriented antennas. Additionally, the focus is frequently on tracking single objects. For the 3D tracking of bats in large-scale wind parks with maximum heights of up to 200 m, X-band marine radar systems are more suitable [25, 34].

A further challenge of marine radar studies is the comparability of the measurements, which is so far ignored in most of the published studies [25]. For comparability of the studies, radar calibration is very important to calculate a theoretical detection volume for the studied objects [25, 28, 32], whereas the determination of their maximum detection range [27, 35] is only an intermediate step in the 3D volume calculation. Accurate calibration is required to calculate object densities defined as counted objects per space, or to calculate mean traffic rates, derived from ornithological studies [28, 36]. Radar detection of bats in an onshore environment is also challenging, because of ground clutter, which is particularly difficult to avoid. It is impossible in certain regions to distinguish weak echoes reflected by bats from interfering signals, resulting in a smaller detection volume.

**Fig 1:**
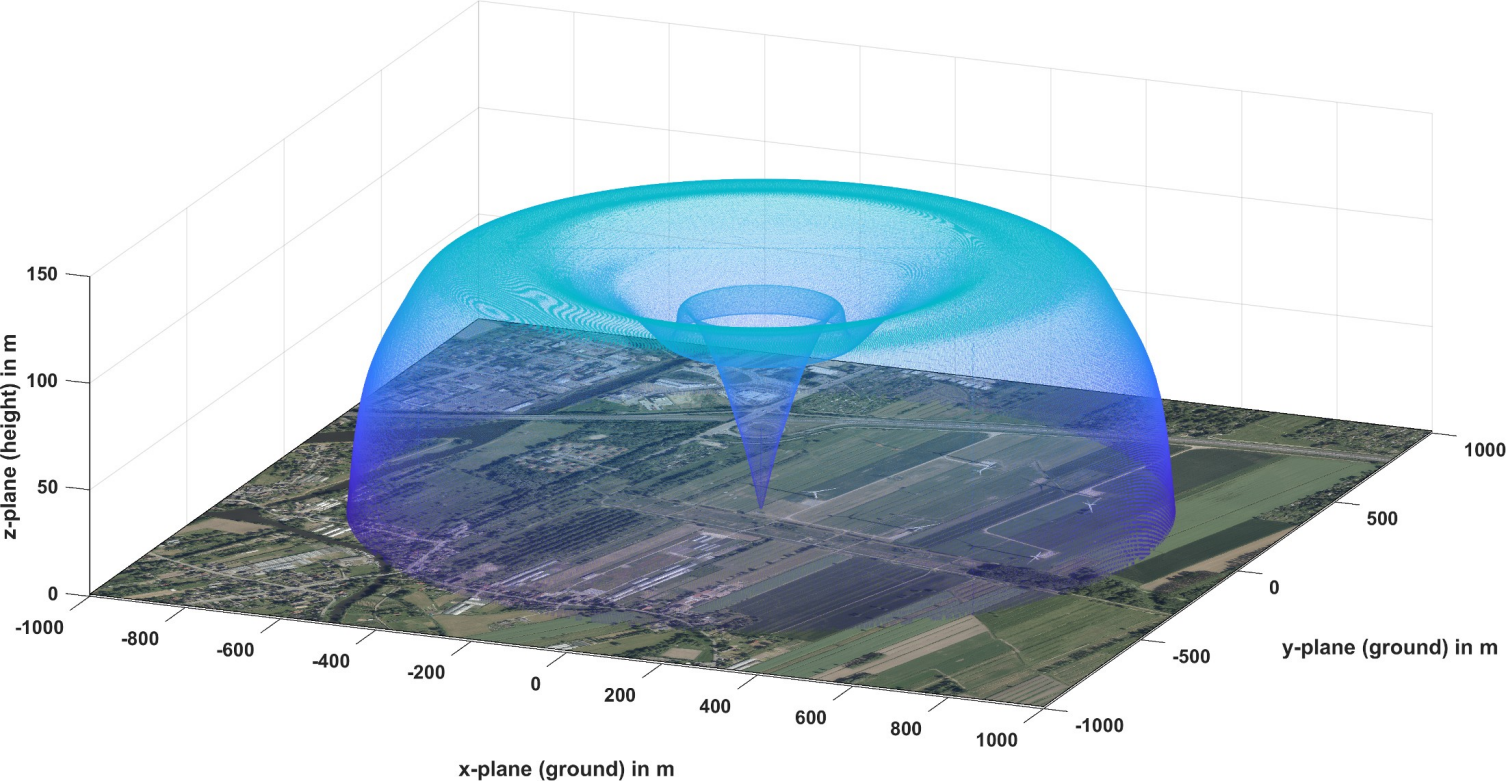
Theoretical 3D spatial detection volume example of a Furuno FAR 2117 marine radar for bat-sized objects (RCS of 12.7 cm²) without influence from clutter. In the satellite image [37] of the wind park and surroundings the radar is positioned in the origin, covering 5 turbines in horizontal dimension, but not fully covering their height up to 180 m.

The objective of this paper is to present an analytical spatial detection volume model of a commercially available inexpensive pulse radar for 3D spatial detection of bat-sized objects to track their flight paths. For visualization, an example of a theoretical detection volume for bat-sized objects is shown in Fig 1, neglecting effects from ground clutter. In total six different input parameter of the model were defined, divided into two superordinate categories:

- Radar calibration data (radar specifications, radar cross section and the minimum detectable signal power)
- Radar image information (the individual study environment including landscape parameters, e.g. clutter).

This model is a novel approach for the determination of the 3D detection volume with a marine pulse radar system and can easily be applied to other studies with similar radar systems and other objects to be studied. Furthermore, only standardized data is utilized, without any modification of the radar system itself.

Radar calibration was performed each time after warm-up. Calibration data for the maximum detection range of our radar for bat sized objects is neither provided by the radar manufacturer, nor available in the literature [25]. The detection range calibration is depending on the radar cross section (RCS) of the object under investigation, the selected radar settings and definitions for the radar image analysis procedure.

Therefore, experiments with a self-developed bat model and additional shapes to provide first estimates of bats RCS and the minimum detectable signal power of the radar were part of this study. An analysis procedure is proposed, providing a conservative detection threshold for bats. It is assumed that the detection threshold is sufficient for the further analysis of automatic tracking algorithms [16, 17].

To optimize the spatial detection volume by minimizing ground clutter, a practical implementation of a physical fence, a so-called clutter shielding fence (CSF), shielding the ground directed radiation is presented. The dimensioning of the CSF is based on laws from physical optics and early military radar shielding studies [38, 39]. To incorporate the influence of the CSF in the spatial detection volume model and to analyze its shielding and diffraction characteristics, a simulation model of the CSF was developed and experimentally verified. Since the entire radar beam is affected by the clutter, the detection range and the detection height of the simulation allows for the comparison of different geometrical setups leading to an optimized CSF. Optimized detection and tracking volumes and increased visibility of the radar inside the wind park can be visualized. Finally, this paper presents recommendations of the optimized settings for a practical pulse radar to study bats in onshore wind parks.

## Material and methods

### Radar setup and study site

The commercially available marine X-band Furuno FAR 2117 pulse radar chosen for this study consists of a magnetron based power-oscillator-transmitter with a peak output power of 12 kW fed into a Furuno XN24AF slotted waveguide antenna, rotating in the horizontal plane with a speed of 24 min^-1^ [40]. Table 1 lists an overview of the radar processor specification and configuration applicable to all measurements presented in this paper.

**Table 1:** Radar specification and hardware parameter for radiation measurements.

**Furuno FAR 2117 radar processor configuration**			
**Specification**	**Configuration**	**Specification**	**Configuration**
Range	1,5 km	Echo Average	Off
Pulse length τ	0,07 μs	Noise Rejection	Off
Pulse repetition freq. PRF	3000 Hz	Auto Sensitivity Time Control	Off
Interference Rejection	Off	Auto Rain	Off
Echo Stretch	Off	Video Contrast	2-B
**Furuno FAR 2117 system specification**			
**Specification**	**Configuration**	**Specification**	**Configuration**
Pulse Power P_t_	12 kW	Antenna Gain g (max.)	31.6 dB
Frequency f	9410 MHz		
**Hardware for radiation measurements**			
**Device**		**Type/Specification**	
Power Meter		Agilent E4418B	
Power Sensor		Agilent E9300A	
Horn Antenna		gain = 15,5 dBi, manufacturer: Procon	

For all measurements provided, the built-in software clutter reduction features were disabled. The utilized pulse length and pulse repetition frequency limit the theoretical range resolution to 10.5 m, while the angular resolution is highly dependent on the distance between the radar unit and the target, allowing for a resolution of approximately 20 m of small targets in range. The digitized radar image delivers an even higher resolution of 3.027 m per pixel at set 1.5 km range in both horizontal X and Y directions. For image extraction in this study, the digital reference signal, visible on the plan position indicator (PPI), is sampled with a digital video interface often referred to as frame grabber (model: Epiphan DVI2USB 3.0).

As the magnetron degrades over time and the receiver unit is sensitive to environmental conditions [18] a calibration procedure must be regularly performed. Therefore, the gain and the tuning frequency were manually adjusted before each session to achieve comparable results [40, 41]. The selectable gain was adjusted so that the receiver noise was just beginning to appear. At this point no more than approximately 75 noise blips (single echoes with small spatial extension) appeared on the PPI.

All studies were conducted in a North German wind park near Hamburg-Curslack, consisting of five turbines with a nacelle height of 120 m. The landscape inside the wind park consists of farmland (mostly pasture) with many small ditches, bushes and a few isolated trees. Clear visibility from radar at eye height into the wind park is provided. In close proximity to the wind park more complex structures such as woods, roads and buildings can be found.

### Analytical model of spatial detection volume

When analyzing bat echoes, the number of echoes or tracks in relationship to the detection volume (e.g. density) is very important to make different studies comparable. Therefore, we developed a 3D model simulating the detection boundaries of height and distance for objects with a defined RCS and given pulse radar parameter. To describe our simulation model we start with the standard radar equation given by [18]:

$$R_{m}{}_{ax}=\sqrt[{4}]{\frac{P_{t}G^{2}\lambda^{2}\sigma }{{\left(4\pi \right)}^{3}P_{min}}}$$ (1)

R_max_ is the maximum detection range, P_t_ is the transmitter power, G is the antenna gain, λ is the wavelength, σ is the RCS and P_min_ is the minimal detectable signal power. To obtain the receiving signal power P_r_ for an object with defined RCS at range R in 3D space we substitute P_min_ with P_r_ and R_max_ with R and rearrange Eq (1):

$$P_{r}=\frac{P_{t}G^{2}\lambda^{2}\sigma }{R^{4}{\left(4\pi \right)}^{3}}$$ (2)

In addition to the physical radar detection parameters described by the variables in Eq (2), clutter has a great influence on the successful detection of small objects. We therefore consider clutter causing landscape parameters of the radar images in our spatial detection volume model as an input parameter.

#### Inputs of the analytical model of spatial detection volume.

1. The radar equation, rewritten to simulate P_r_ (Eq (2)) and fixed values of P_t_ and λ (see Table 1).
2. The vertical antenna diagram of the radar antenna to consider the vertical gain distribution: G(ϕ), where ϕ is the elevation angle (available from radar manufacturer).
3. The RCS σ of the object of interest.
4. The minimum detectable signal power P_min_ corresponding to the defined analysis procedure (described in the following section).
5. A radar image (or the defined image section) to be analyzed. Based on the resolution and range settings, pixel dimensions A_px_ must be calculated (A_px_ = 9.16 m² for this study).
6. A clutter threshold based on the echo resolution to define the clutter signal power (The Furuno FAR 21x7 radar possesses an echo signal resolution of 32 levels, where each level is represented as an RGB (red, green and blue) value of the RGB color space).

### Radar calibration

#### Determination of bats radar cross section

To determine the RCS of the largest bat (*Nyctalus noctula*) found in this wind park, experiments with an artificial bat model and further test objects for comparative purposes were performed. A detailed description of the bat modelling and the test objects is given in the RCS modelling section. Based on maximum detection range experiments with the bat model in the wind park, the related RCS was calculated by rewriting the standard radar Eq (1) [18]:

$$\sigma =\frac{R_{max}{}^{4}{\left(4\pi \right)}^{3}P_{min}}{P_{t}G^{2}\lambda^{2}}$$ (3)

Where P_t,_ G and λ are constants, while only P_min_, accumulating all losses (e.g. due to noise), must be determined separately (described in the next section).

**Fig 2:**
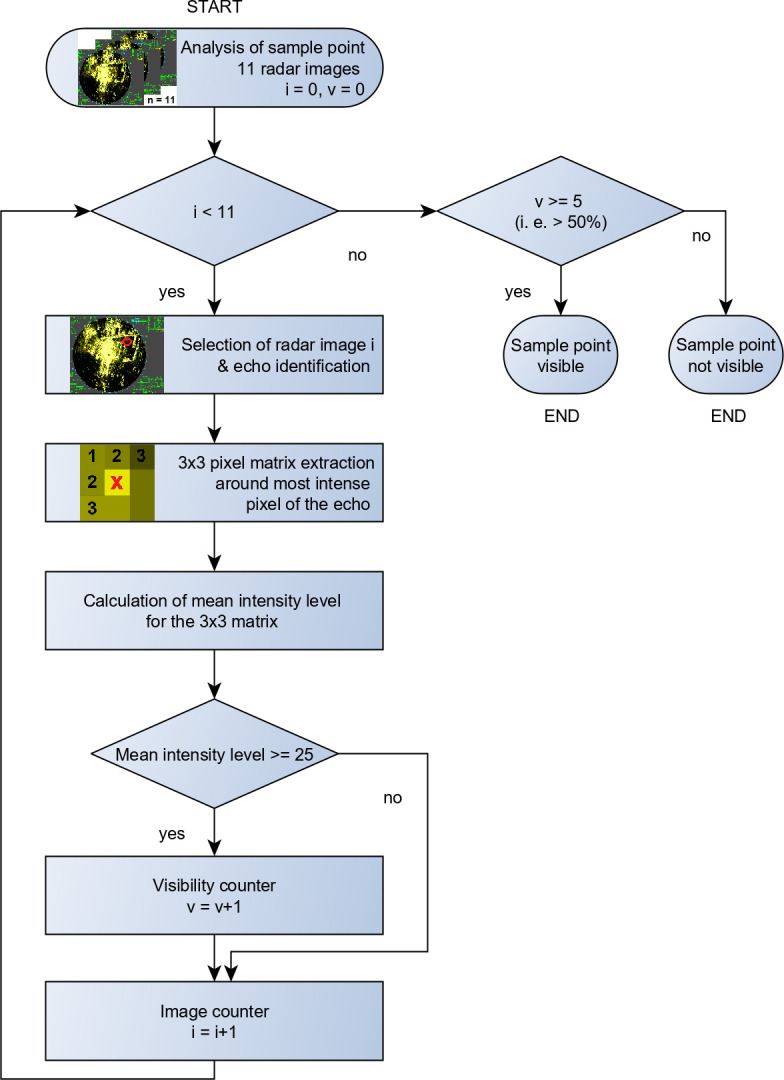
Analysis procedure for radar images. Each image of a set of 11 consecutive images (image counter “i”) at individual sample points is compared against the mean intensity level of 25 (where 25 is a unitless radar intensity level based on the RGB value). Brighter samples cause the visibility counter “v” to increment. After each set of images is executed, the visibility counter “v” is compared against a threshold value of 5. The sample point is defined “visible” when “v” exceeds the value of 5 (>50%).

Sample points were placed between 350 m and 950 m in steps of 150 m from the radar and chosen to be in areas with minimum background clutter. At each sample point a set of 11 consecutive radar images were captured at a frequency of 0.4 Hz. This image set together with the visibility definition (see below) between consecutive radar scans provides sufficient data to compensate echo fluctuations. The odd number of 11 was chosen experimentally after a series of trials with different visibility definitions (definition see below). The bat model was attached to a thin string dangling between two poles at a height of 2.5 m, which is equal to the antenna height. As echoes are fluctuating and represented with a resolution of 32 levels (unitless, visualized in a different RGB value per pixel, see also input 6 of the volume model) the analysis procedure, described in detail in the flow chart in Fig 2 was applied. If the echoes in the single radar images meet certain thresholds (defined in Fig 2) they are counted visible. The sample point is determined as “visible”, when more than 50% of all images meet the threshold criteria, so that at least one strong echo in every second radar scan remains. The selected thresholds (e.g. matrix dimension and the mean intensity level) were defined after several iterations in manual analyses of radar images with fluctuating clutter and provide a very conservative threshold for object identification (see an example non-manipulated radar image in supporting information S1 Fig).

#### Determination of the minimum detectable signal power

The minimum detectable signal power P_min_ underlays statistical fluctuations of the signal to noise ratio and individual losses of the radar system [18]. Recommendations for a theoretically calculation of P_min_ are given by [32]. Since the theoretical parameters can vary between different radar systems, we followed a more practical approach. We recalculated P_min_ from a field experiment with an object of known RCS. Therefore, we experimentally determined R_max_ of the object with known RCS using the analysis procedure, similar to the RCS determination method of the bat model. With this result, the known RCS and rewriting Eq (1), P_min_ could be calculated.

**Fig 3:**
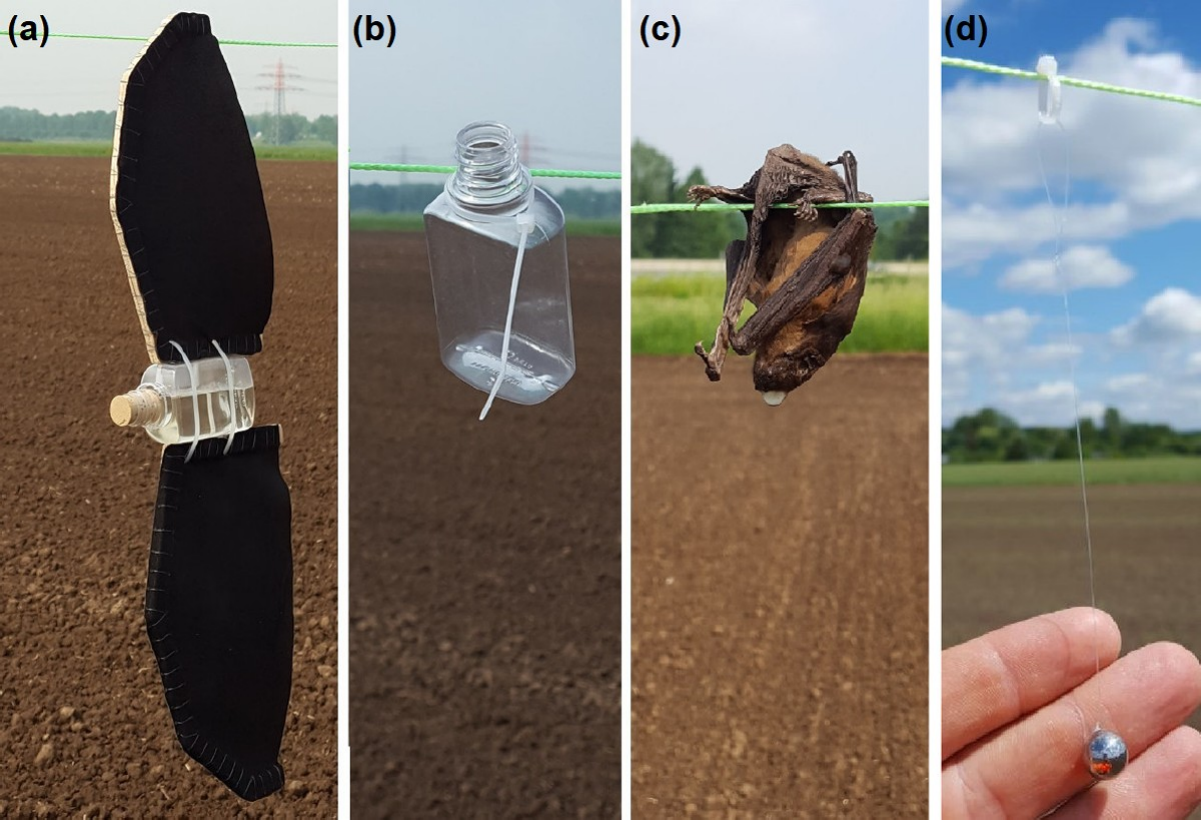
Test objects to experimentally determine the maximum radar detection range. (a) reference bat model with water filled PET bottle as torso (water sphere simplification) and leather wings, (b) empty water bottle (water filled bottle without wings is not displayed), (c) dead bat, (d) metal sphere (bearing steel 100Cr6) for calculation of minimum signal power.

An ideal test object with known RCS is a perfectly conducting sphere, e.g. a metal sphere, where the RCS is independent from the physical appearance [41], but a function of its cross-sectional area and the wavelength λ [19]. Therefore, a solid metal sphere from an industrial bearing with a diameter of 1 cm and a radar cross section of σ_1cm_ = 2.8 cm² was used in the experiments as depicted in Fig 3D. The calculation of a perfectly conducting sphere allows simplified calculations compared to calculations of more complex shaped objects. The calculation of several normalized shapes can be found in e.g. [42], allowing the calculation of the absolute cross section σ_sphere_ of a metal sphere for a given radius r to:

$$\sigma_{sphere}=f_{r}{}_{atio}k\pi r^{2}$$ (4)

where k is the scattering factor and f_ratio_ the diffraction.

The influence of diffraction is depending on the ratio of r over λ, which is ≈1 in the optical region. The scattering factor corresponds to the dielectric constant ε_r_ of the material. It is approximately 1 for a perfectly conducting sphere [43]. A sphere with a circumference smaller than approximately ten times the wavelength is leaving the optical region and experiences a huge variation in their RCS due to Mie-Scattering (e.g. diffraction around the sphere leads to positive and negative interference with direct reflected radiation). For smaller circumferences than the wavelength, the cross section drops due to Raleigh-Scattering [19, 44].

#### Radar cross section modelling of bat-sized objects

For modelling small size objects such as bats, a concept invented by radar ornithologists was utilized. Since the RCS varies depending on the material, appearance, size, wavelength and polarization [41] making theoretical calculations difficult, Houghton and Schaefer simplified the RCS of birds to water spheres and spheroids [20, 21, 43]. Hereby the bird’s torso, mainly consisting of water was understood as being the main reflector, whilst the bird's feathers are negligible. For the calculation of a water spheres RCS, Eq (4) can be applied, leading to similar variations due to diffraction effects. However, the scattering factor is now adjusted to k = 0.56 as suggested by Eastwood [19].

As a reference test sample, the largest endangered bat found in the wind park (*Nyctalus noctula*) was modelled with a torso weight of 30 g, torso length of 8 cm. and a wingspan of 40 cm [45]. In contrast to the concept from ornithologists, wings were also considered and attached to the model, because an influence on the RCS caused by the different material characteristics of bat wings is assumed. Based on the water sphere simplification, the torso consists of a tiny cuboid PET plastic bottle filled with 30 ml of water (density of 1 g/ml). Measurements with a calibrated power meter (see specifications listed in Table 1) have shown that the influence of the plastic material can be neglected. Calculating the RCS for a water sphere with a volume of 30 ml (corresponding to a radius of 19.3 cm) in the optical region, Eq (4) with k = 0.56 and f_ratio_ = 1 results in an RCS of σ_optical_ = 6.65 cm². Including Mie-scattering the RCS rises to σ_Mie1_ ≈ 7.5 cm², which is equal to the result found by [46] and close to the result found by [42] for the RCS of ≈ 8.1 cm². The wings of the bat model consist of two 0.4 mm thick leather slices with a total area of 234 cm² and are glued to a thin wooden frame. The underlying assumption of this task was that leather has similar properties to bats real wings. The assembled model is shown in Fig 3A. The shape of the wings were modelled according to pictures and drawings from [47].

Since the water content seemed to have the biggest influence on reflectivity, the water content of the artificial wings was compared to the water content of wings from a dead bat. Mass related relative water content of the artificial wing was 9.8%, while the wing of the bat specimen contained 17.7% water. Comparing the weights of the wings, the artificial wing had approximately double the weight whilst covering the same area (total weight per artificial wing was 6.32 g). For comparison, the RCS of the single torso (bottle with and without water, see Fig 3B) was also assessed in the RCS experiments described above. Furthermore, a dead bat with a weight of 14.7 g served for comparison as the fourth sample (see Fig 3C).

### Optimization of the spatial detection volume

#### Design and calculation of a CSF

A typical marine pulse radar with a slotted waveguide antenna has a vertical antenna gain distribution of approximately ± 10° (for the -3 dB region) around the horizontal plane. Pulse radar onshore scanning typically results in strong reflections from surface structure, so called land clutter [48]. To optimize the spatial detection volume, a physically constructed reflective shield (CSF) was developed and simulated to be incorporated in the volume model. CSF are used in military applications since the sixties [38, 39] and are still commonly used today, for example in earth and space science radars to reduce land clutter [49]. The general idea is to have a fully opaque fence with respect to the utilized wavelength, positioned at a fixed distance to the radar antenna covering an area of several square meters [38].

**Fig 4:**
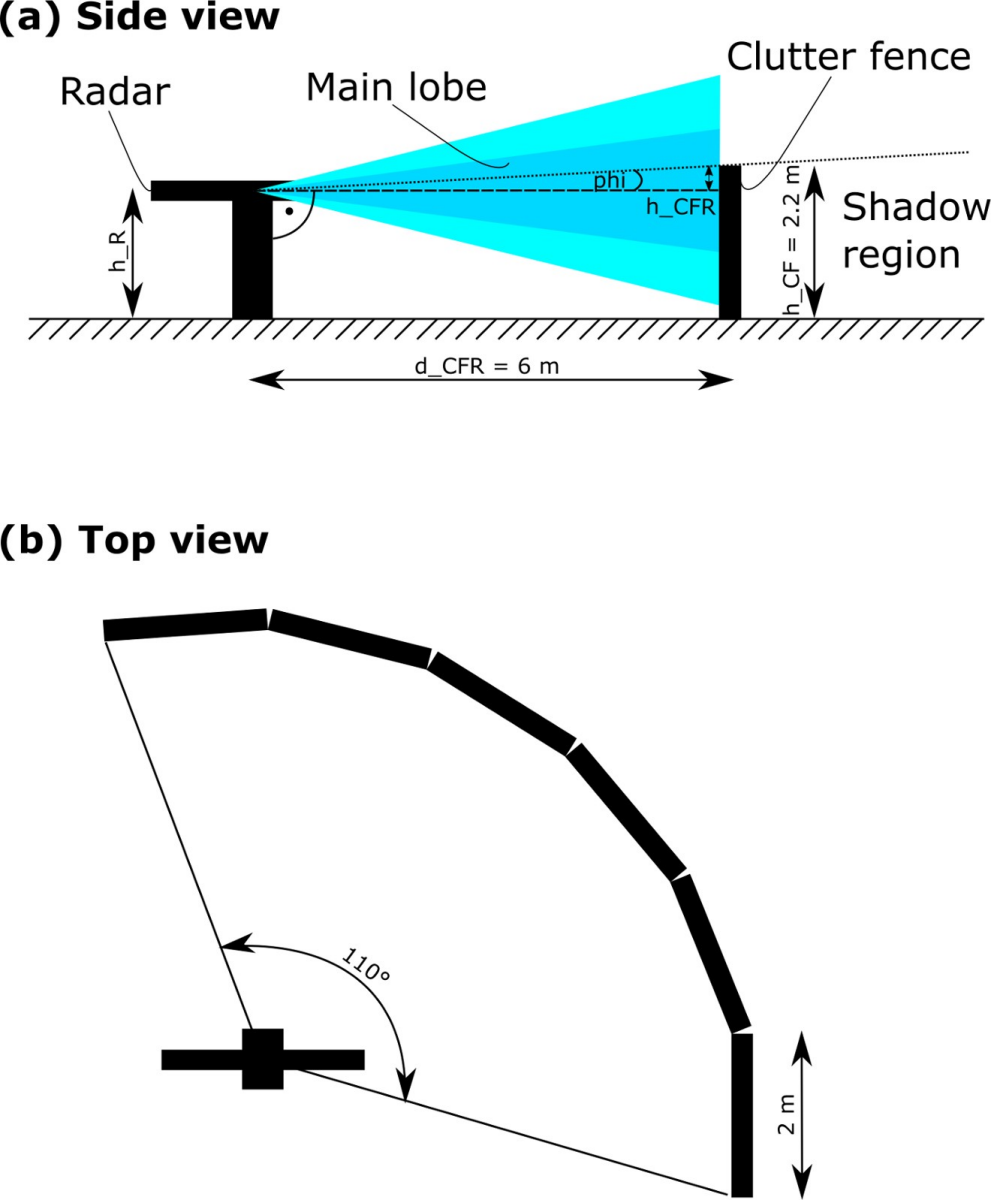
Radar and CSF setup. a) Side view showing the shadow region. b) The CSF of 2.2 m height covers an angular section of approx. 110° around the radar and shields the lower part of the simplified radiation (drawing is not true to scale).

To shield the X-Band radar waves (λ = 3.1 cm) a stainless-steel wire fence with a mesh spacing of 1 mm was assembled. Our mesh wire fence is relatively light weight (approximately 1 kg/m²) and permeable to wind, reducing wind pressure on the CSF. Initial measurements of the wire fence showed one-way attenuation in the range of -20 to -25 dB indicating that most of the radiation is reflected by the wire fence. The wire fence is mounted on rectangular wooden frames, which are arranged in a polygon around the antenna as depicted in Fig 4. The fence consists of six sides with a width of 2 m covering approximately 110°. The inner polygon radius is 5.99 m, while the outer radius is 6.08 m leading to an average radius of 6 m (d_CFR_). This distance was chosen based on preliminary shielding effectiveness assumptions [38]. The fence height (h_CF_) was chosen to be 2.2 m, as most clutter is assumed to be received from ground level, whilst the radar antenna height (h_R_, up to the center of the antenna) is adjustable in order to find the optimum height setting in combination with the fixed height of the CSF.

The diffraction induced by the upper edge of the fence effects the receiving signal power P_r_ in Eq (2) and therefore the shadow region behind the CSF cannot be ignored (shown below the dotted line in Fig 4A). Based on data from [38] our CSF setup with h_CFR_ = 2.2 m, h_R_ = 2 m and d_CFR_ = 6 m yields a relative one way clutter suppression of approximately 10 dB. This accounts for objects ranging in height from 0 to h_R_ behind the CSF independent from their distance.

**Fig 5:**
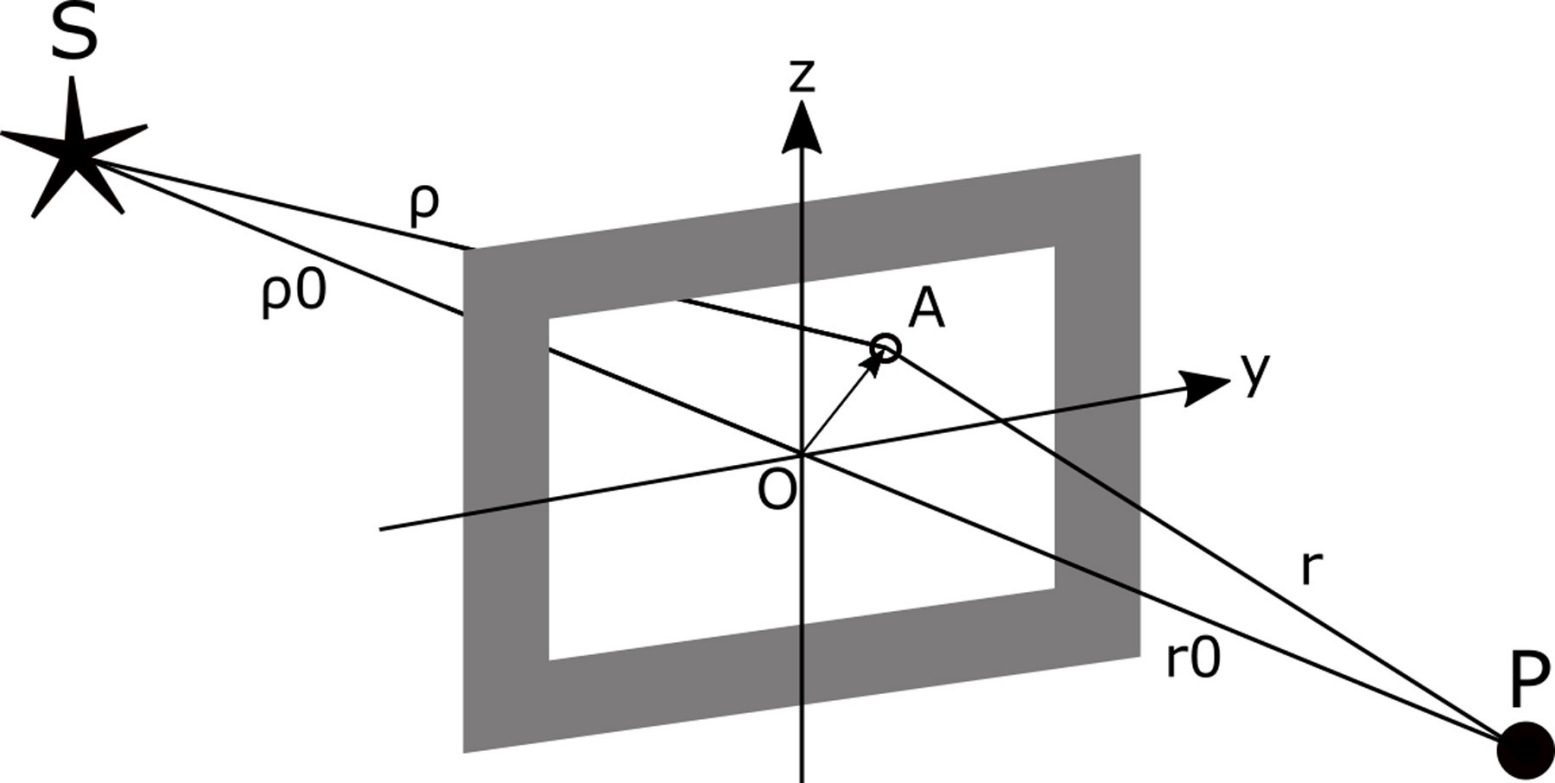
Fresnel diffraction at a rectangular hole, adapted from [51]. The intensity of the radiation emitted from source S and received at point P through a rectangular hole is interfered by all points inside, depended on their geometrical description, here visualized for a single point A connected by the indirect radiation lines ρ, r.

*Theory of diffraction at the fence edge*. Wave diffraction is a well known phenomenon in optics and is defined as the deviation of wave propagation behind an obstacle, such as the edge of a CSF or a hole from its original geometrical direction [50]. Two major mathematical models to describe this phenomenon exist, namely Fresnel diffraction and Fraunhofer diffraction, where the latter is a simplified version of Fresnel diffraction and only valid for planar waves in the far-field. Because of the infinite dimensions in y and z-direction shown in Fig 5, we cannot apply Fraunhofer’s planar wave simplification to our CSF edge. Instead, we apply the mathematical sharp edge Fresnel diffraction model for spherical waves.

In the following section, an overview of the most important diffraction principles [51] is given to finally modify the analytical model of spatial detection volume. Fresnel diffraction at a rectangular aperture, shown in Fig 5, describes the intensity of the radiation emitted from source S received at point P (direct lines of ρ_0_, r_0_), which is interfered with secondary radiation coming from all points inside the hole (indirect lines of ρ and r). The interference is determined by the geometrical setup of the hole between S and P, while sharp edge diffraction is simply this hole with infinite dimensions on the Y-axis and the positive Z-axis.

The intensity I_P_ at point P for a rectangular hole can be calculated with the help of the Fresnel integrals C and S:

$$I_{P}=\frac{I_{0}}{4}\{{\left[C(u_{2})-C(u_{1})\right]}^{2}+{\left[S(u_{2})-S(u_{1})\right]}^{2}\}\times \{{\left[C(v_{2})-C(v_{1})\right]}^{2}+{\left[S(v_{2})-S(v_{1})\right]}^{2}\}$$ (5)

Where I_0_ is the intensity of the undisturbed radiation, u and v are dimensionless variables, describing geometrically the hole in the y- and z-axis and incorporating the wavelength of the radiation:

$$u=y\sqrt{\frac{2(\rho_{0}+r_{0})}{\lambda \rho_{0}r_{0}}}$$ (6)

$$v=z\sqrt{\frac{2(\rho_{0}+r_{0})}{\lambda \rho_{0}r_{0}}}$$ (7)

When the dimensions of the hole are small in relation to ρ_0_ and r_0_ it can be assumed, that the Fresnel obliquity factor equals one, therefore:

$$\frac{1}{\rho r}\approx \frac{1}{\rho_{0}r_{0}}$$ (8)

For the sharp edge diffraction of the CSF u_2_ = v_2_ = ∞ and u_1_ = -∞, Eq (5) simplifies to:

$$I_{P}=\frac{I_{0}}{2}\left\{{\left[\frac{1}{2}-C(v_{1})\right]}^{2}+{\left[\frac{1}{2}-S(v_{1})\right]}^{2}\right\}$$ (9)

where only the calculation v_1_ (Eq (7)) in the vertical z-axis is required.

#### Modification of the spatial detection volume model

The influence of the diffraction is considered by multiplying the receiving signal power P_r_ from Eq (2) in the analytical volume model (e.g. input 1) with the normalized intensity I_P_ of Eq (9). This yields to the modified receiving signal power P_rm_:

$$P_{r}m=P_{r}I_{P}^{2}$$ (10)

Where the intensity I_P_ appears in the send and received signal. Because Eq (9) is based on spherical waves, we can assume that the main beam of the antenna always focuses on the CSF edge and further, that for lower elevation angles the antenna gain is relatively constant. This is the case for standard marine slot antennas, whilst for higher angles, the diffraction has only little influence and deviations in the calculations are therefore negligible.

#### Experimental determination of the optimal detection volume

In practical experiments, radar images were captured at different heights of the radar antenna in relation to the CSF (h_CFR_ in Fig 4). The height was varied in range between -0.096 m to 0.91 m by adjusting the radar antenna height (h_R_; antenna exceeds the edge of the fence at negative values), whilst the position of the CSF remained unchanged. These adjustments lead directly to a variation of the geometrical dimensions in the variable v of Eqs (7) and (9) changing the shielding characteristics of the CSF. For absolute value comparison, a radar image without CSF was also captured. These images served as a 5^th^ input of the analytical model to calculate the corresponding detection volume. Thereby only the radar image section inside the CSF coverage (see Fig 8) is considered in the calculation, as the fence is not covering 360°. Input 3 and 4 (σ = 12.7 cm², P_min_ = -74 dBm) were selected based on the calibration results. By varying h_CFR_ and the clutter threshold as 6^th^ input of the analytical model from a level of 0 to 24 with a step size of 8, an optimal detection volume was determined.

### Simulation of the optimized spatial detection volume

In order to compute the spatial detection volume based on the analytical model inputs, a computer simulation was programmed in Python. Thereby two steps are combined, each providing detection information in two dimensions, to finally calculate the 3D detection volume. When optimization is applied, the first step is divided into two subsections.

#### 1a. Simple detection range simulation

In the first step the detection height over distance is calculated by incorporating radar calibration parameters.

#### 1b. Advanced detection range simulation

Adjustment of the first step by incorporating the equations describing the optimization effects from the CSF.

#### 2. Horizontal visibility simulation

In the second step landscape parameters from the radar image and clutter threshold settings describing the visibility in the horizontal plane are considered.

To confirm the reliability of the ground clutter shield in simulation 1b, two different verifications were performed. First, the visibility of bat sized objects in the transition to the geometrical shadow region of the CSF (see Fig 4) was evaluated in a practical field test. Second, the influence on the radar beam due to Fresnel diffraction was evaluated with radiation measurements in the most affected region (measurement hardware, see Table 1) behind the CSF. Both outcomes were compared with simulation results.

## Results and discussion

### Bats radar cross section and minimum detectable signal power

**Fig 6:**
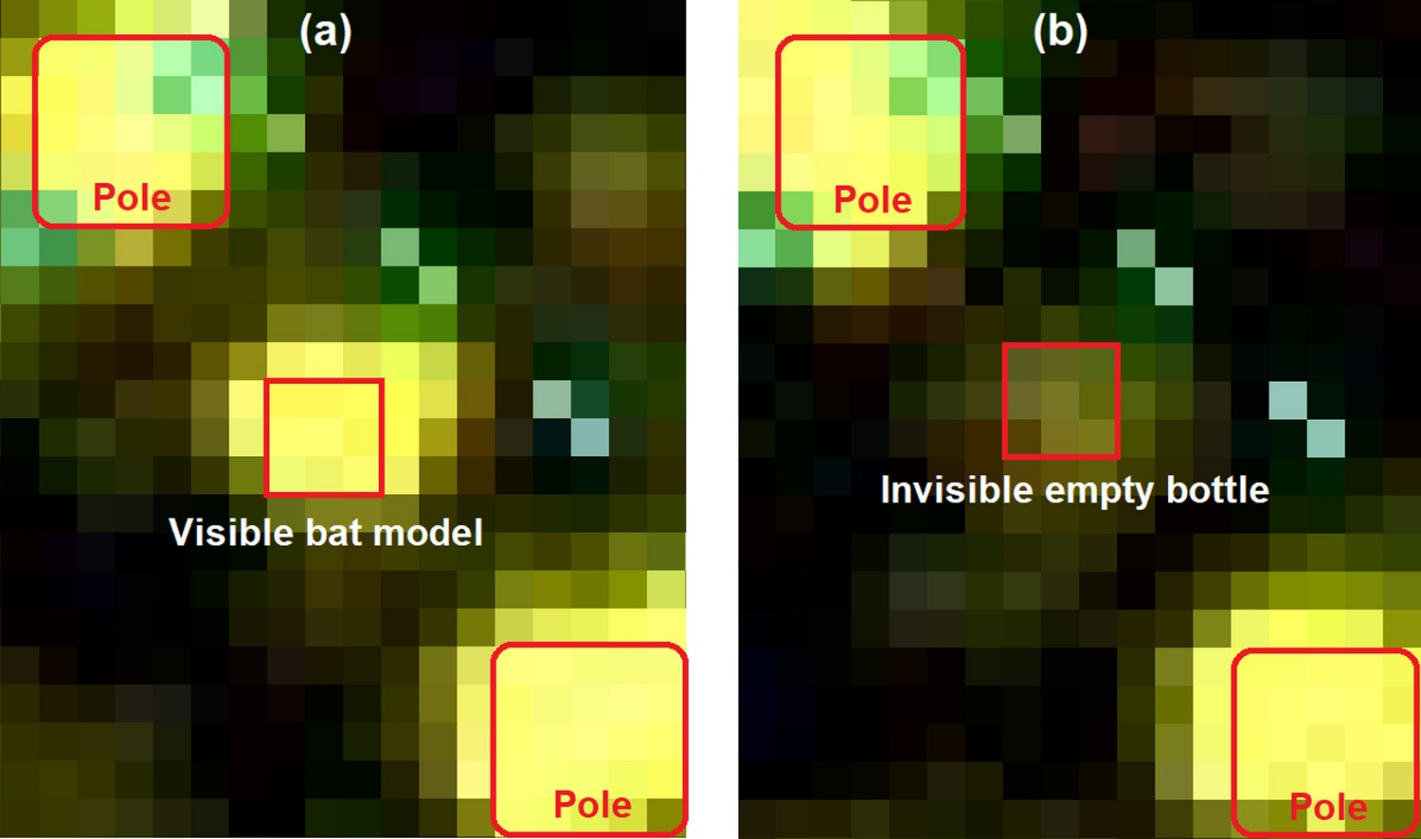
Echo examples of the detection range experiments. (a) shows the visible bat model at a mean intensity level of 31 and (b) an invisible empty bottle at a mean intensity level of 18, both at 650 m distance from the radar and marked with the 3x3 analysis pixel matrix around the brightest pixel. The poles of the measurement setup clearly surround the test object, while green pixel result from radar range markers.

Fig 6 shows a visible and an invisible echo signature (two different radar scans) as example from the detection range experiments, which served as basis for the bat models RCS calculation. The echoes of the poles from the experimental setup are also clearly visible. The visibility evaluation is shown in column 2 to 6 of Table 2. Here the measured results for 5 different distances from the radar are listed. For each distance measurement, the echoes of 11 radar images were analyzed based on the previously described procedure not only for the bat model, but also for the comparative test objects.

**Table 2:** Detection ranges of the bat model and the additional test samples.

	Visible echoes per distance from radar				
	350 m	500 m	650 m	800 m	950 m
**Bat model**	11/11	10/11	9/11	6/11	0/11
**Water bottle**	9/11	0/11	10/11	0/11	0/11
**Empty bottle**	10/11	0/11	0/11	0/11	0/11
**Dead bat**	10/11	4/11	11/11	0/11	0/11

At each distance shown in row 2, a set of 11 consecutive radar images (echoes) for every test object was evaluated according to the defined analysis procedure. The table lists the number of visible echoes found in respect to the 11 consecutive radar images.

The bat model was visible up to a distance of 800 m. At 950 m distance, none of the objects were visible. The water filled bottle and the dead bat had a very similar echo signature; but were only visible up to a distance of 650 m. This result proofs the RCS water sphere simplification assumed for birds without wings, as the dead bat had its wings closed. Since the bat model had open wings and the same water bottle as torso and was visible at greater distance, an influence of the wings could be identified, which is in line with the results from [52]. Interestingly, this result is contrary to that shown for birds [20], where the authors conclude that bird wings are negligible regarding the RCS. From the biological body structure the contrasting results are reasonable, as feathers from birds include almost no water and consist mostly of the protein keratin [53], while the water content of the wings (9.8%) accounts for the RCS extension of the bat wings.

Based on these measurements, a calculation of the bat model’s RCS, Eq (3) and P_min_ = -74 dBm (value taken from results below) lead to σ_bat_model_ = 12.7 cm². These results are very similar to the results presented by [35, 43, 52]. RCS calculations of the water bottle and the dead bat with a maximum detection range of 650 m result in σ_dead_bat_ = σ_water_bottle_ = 5.5 cm². This is comparable to the theoretical results of 7.5 cm² and 8.1 cm² for a 30 g water sphere evaluated by [46] and [42]. Since the empty bottle was only visible up to a distance of 350 m, it can be concluded that the PET bottle itself has very little influence on the reflection properties of the bat model torso. This would indicate that the RCS of the empty bottle is approximately σ_empty_bottle_ = 0.5 cm².

Against our expectation, the number of visible echoes at 500 m distance of the water bottle and the dead bat was very low. This behavior could be a result of strong signals from a nearby turbine and two trees at the sample point, which may raise the threshold for signal detection in the radar processor in the whole area. The detection threshold at 500 m distance could have been slightly above the echo signal strength from the dead bat and the water bottle, but just below the threshold in case of the bat model (the largest RCS of our samples). As these thresholds are generally unknown at every position, it is recommended to have large clutter free areas around each sample point to make results more comparable. In this study, however, it was very difficult to find appropriate sample points at short ranges (up to approximately 600 m) due to large clutter areas.

In the second detection range experiment, R_max_ of the metal sphere with a diameter of 1 cm was determined to be 550 m, which served as a reference for the calculation of the minimal detectable signal power. Again, the analysis procedure was applied to the data. Solving Eq (1) for the minimal detectable signal power P_min_ leads, for the determined R_max_ = 550 m and the previously calculated RCS of 2.8 cm², to -74 dBm.

The extracted RCS of our models match accurately with the theory as well as with results from similar studies, which further indicates that our experimental results are very precise. However, the minimum detectable signal power is relatively high, compared with other studies [28, 32, 33, 35]. This fact is due to the conservatively selected mean intensity level of 25, defining the visibility of the analysis procedure. The RCS of moving objects such as bats is dependent upon the orientation of the object with respect to the radar beam [18, 19, 41]. In our case the chosen constant RCS can be understood as an average RCS.

### Computation of the spatial detection volume

**Fig 7:**
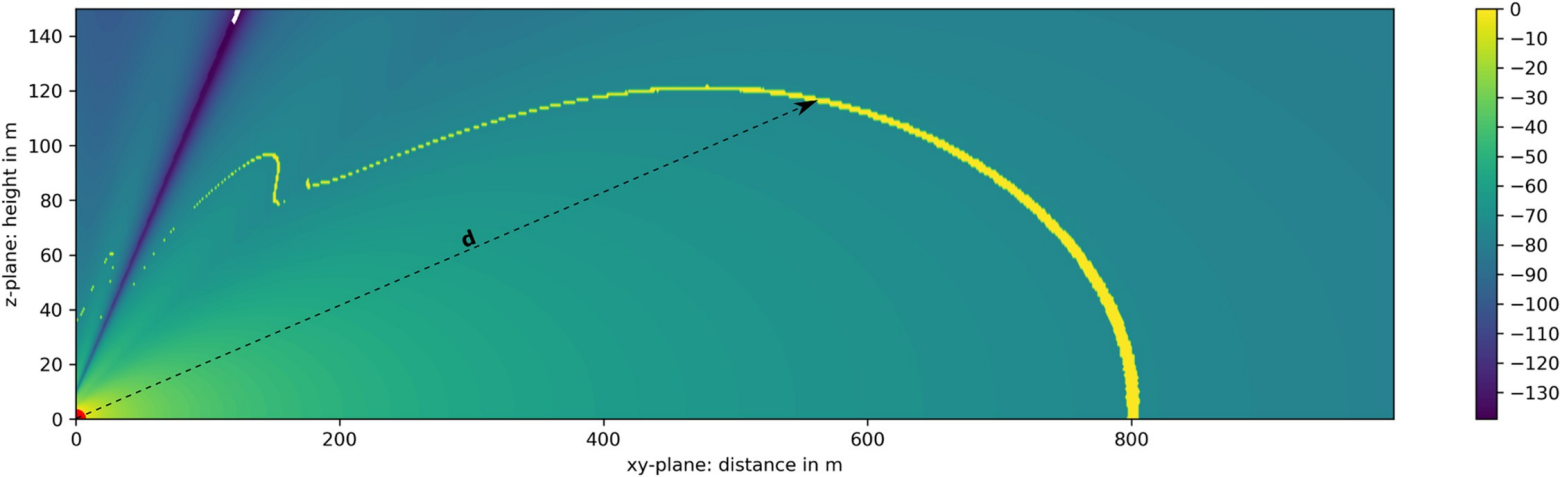
Simple simulation of 2D radar receiving power in dBm and the max. detection range for objects with σ = 12.7 cm². The radar is positioned in the origin indicated in red. Every object inside the yellow boundary (P_min_ = -74 dBm) is detectable. The dotted arrow indicates the detection height for an object at distance d.

In the first step of the volume calculation (1a. Simple detection range simulation), the 2D detection range of an object with a definable RCS (here the bat model with σ = 12.7 cm²) is simulated over distance and height as shown in Fig 7, representing a vertical cross section of Fig 1. The simulation processes inputs 1 to 4 of analytical volume model and calculates the receiving signal power of the defined object in distance from the radar, which is positioned in the origin. With the minimum detectable signal power (here P_min_ = -74 dBm), the detection range was calculated for the bat model (yellow line) and has a maximum of 800 m in distance and of 120 m in height.

**Fig 8:**
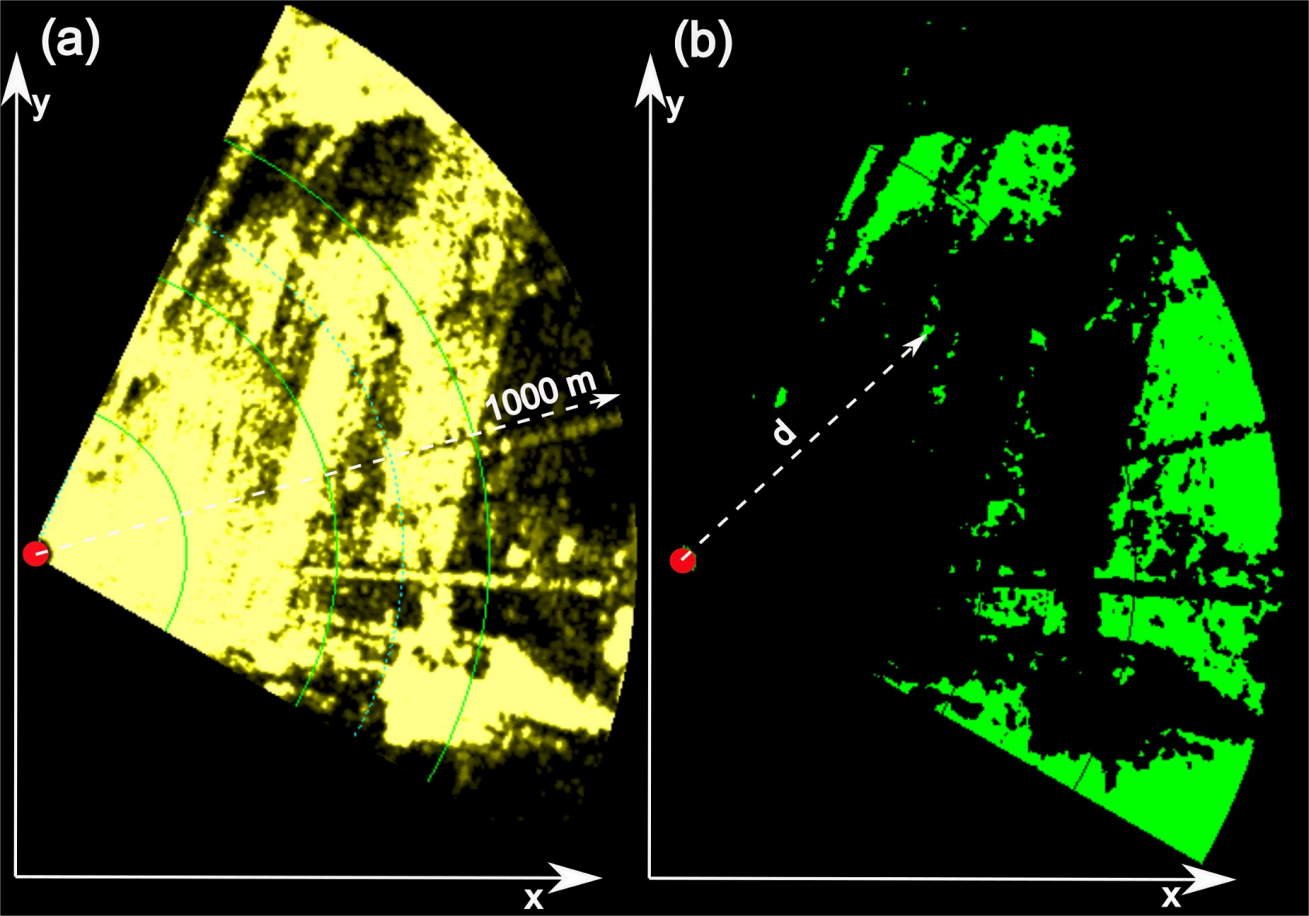
Visibility of a radar image section. In (a) a highly cluttered radar image section from 25° to 120° and a max distance of 1000 m in the xy-plane with various yellow colored clutter intensities is shown (radar is positioned at the red dot). (b) shows the remaining visible regions in green color after reducing clutter below an intensity level of 11 and the distance d to an arbitrary visible pixel p.

In the second step (2. Horizontal visibility simulation), a selectable image of the radar site (input 5, Fig 8A) is scanned to extract visible regions (Fig 8B) based on a set clutter threshold (input 6) in a defined image section in the horizontal plane (e.g. range and angle). With a lower threshold, the green colored visible regions (and therefore the number of green pixel) increase. The example radar image in Fig 8A contains a large amount of clutter of various intensities, where the yellow hue visualizes the signal strength (see input 6 of the volume model for description of the radar resolution).

The two-dimensional output data of the detection range model depicted in Fig 7 and the two-dimensional visible radar image section data depicted in Fig 8B are now combined to compose the final 3D volume model.

The following algorithm is applied to each pixel p shown in Fig 8B:

1. Calculate the distance d from the radar to the visible pixel p
2. Determine the detection height h of pixel p at its distance d (this data is taken from the yellow detection range curve in Fig 7)
3. Calculation of the represented volume of pixel p by multiplying its surface area A_px_ (A_px_ is constant 9.16 m²) with the pixel’s detection height

The overall volume V is the sum of all visible pixel volumes:

$$V=\sum_{p=1}^{n}A_{p}{}_{x}\;h(d_{p})$$ (11)

Where n is the total amount of visible pixel.

**Fig 9:**
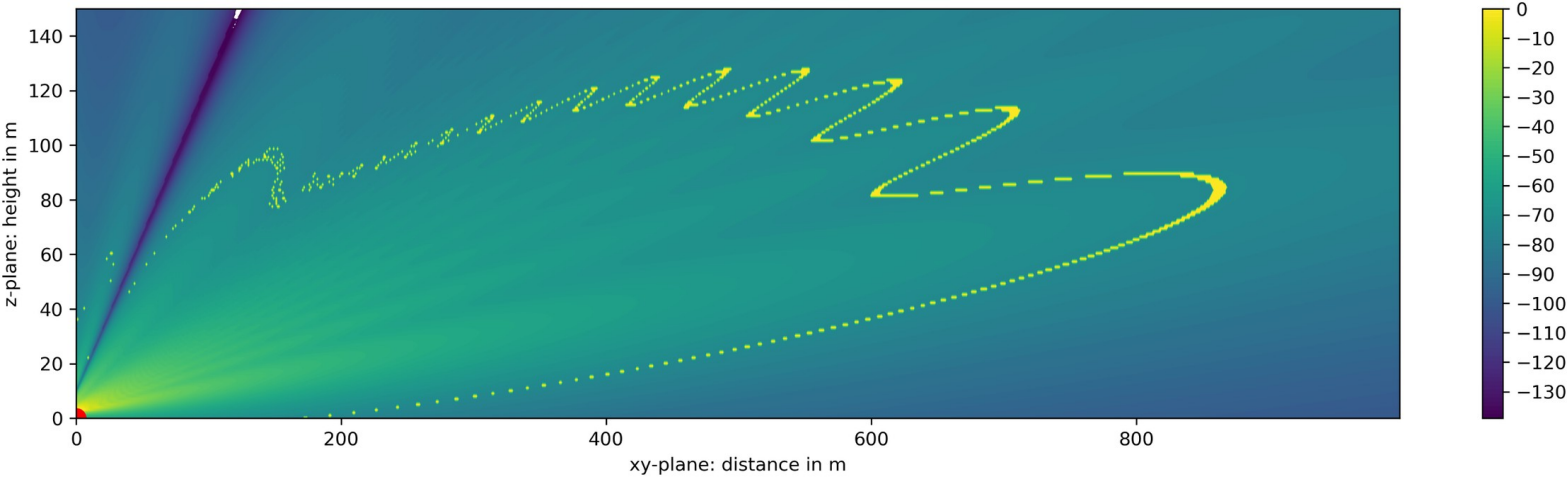
Advanced simulation of 2D radar receiving power in dBm and the max. detection range due to diffraction at a CSF. The Fresnel sharp edge diffraction at the CSF leads to the desired attenuation of ground signal power, whilst for higher elevation angles a modulation resulting in maxima and minima appears. The radar is positioned in the origin indicated by the red dot. Settings: h_R_ = 2 m, h_CF_ = 2.2 m, d_CFR_ = 6 m, σ = 12.7 cm², P_min_ = -74 dBm.

By incorporating the optimization of the CSF, the first simulation step is adjusted with the advanced detection range simulation (1b). Hereby the modifications to input 1, described earlier in Eq (10) are applied in the 2D detection range simulation. The simulation output for a geometrical clutter setup according to the definitions in this study (see CSF design section) is shown in Fig 9. Compared with Figs 7 and 9 shows far less signal power at ground level, minimizing ground clutter at all target distances. The amount of visible pixel in the radar images is increased, the detection volume is enlarged, while the detection range has a raised new lower boundary (lower part of the yellow dotted line rising from 200 m distance).

As a side effect, the modulation of the signal power at higher elevation angles lead to little volume reductions, which are also a result of the Fresnel diffraction. However, the above-mentioned increase of visible pixel predominates very much, optimizing the whole detection volume.

#### Significance of the advanced 2D detection range simulation

The verification experiments follow very well the simulated data of Fig 9 and verify the shadow region (with lower detection boundary) behind the CSF and the visible region up to the second detection range maximum of the modulated detection range curve (yellow curve in Fig 9 inclining from the origin to the 2^nd^ maximum at approximately 700 m distance and 110 m height). With the general existence of the lower detection boundary, the shielding effectiveness in the shadow region is verified. The boundary’s exact position is also an indicator for correctness of the maximum detection range and the absolute signal power of the antennas main beam, which induces the sharp edge diffraction. The modulation at high elevation angles could not be analyzed, as its dimensions in space exceeded the studies possibilities. However, as assumed in the methodology section, the diffraction influence in that region is negligible.

### Optimized radar detection volume for bat detection in onshore wind parks

#### Largest volume optimization

**Fig 10:**
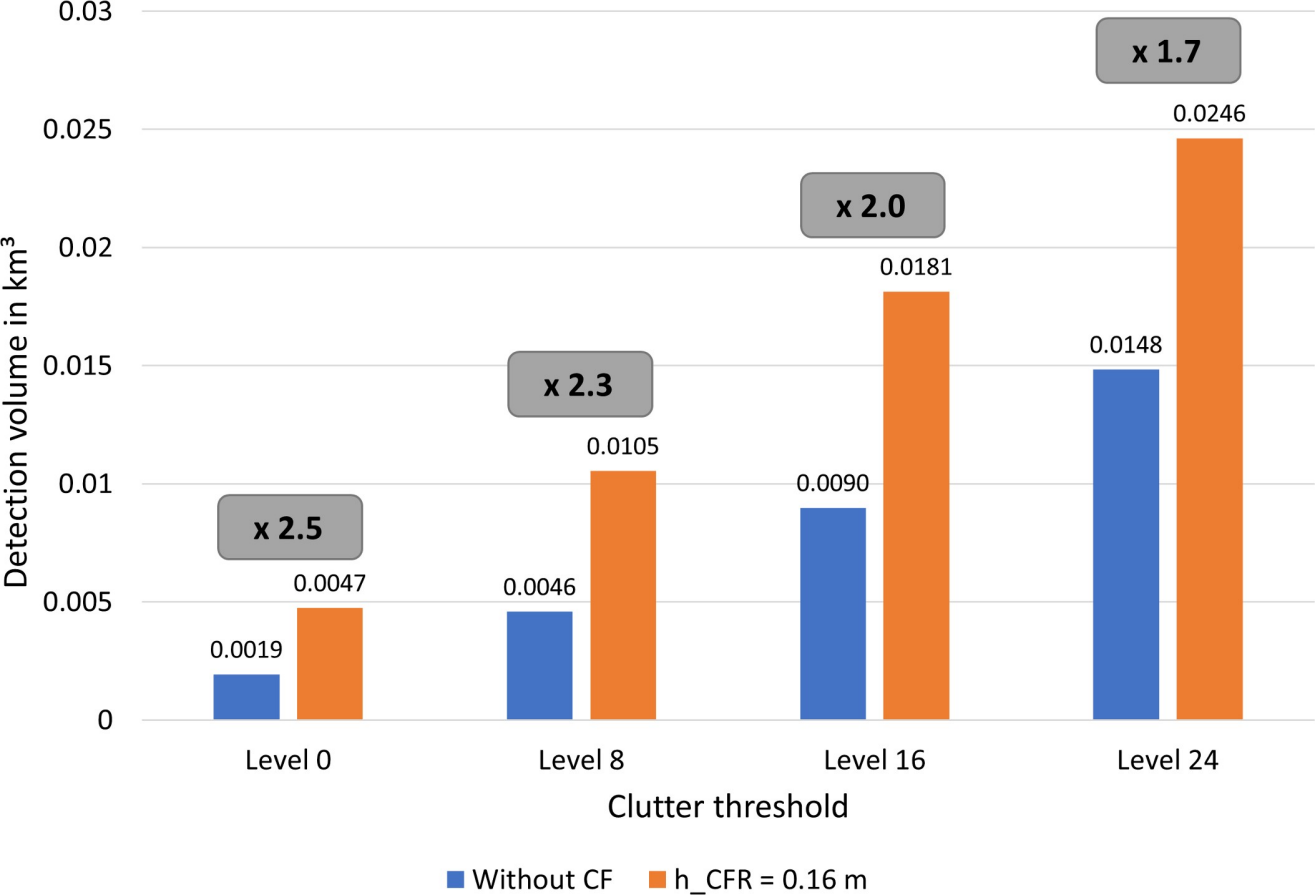
Increase of the radar detection volume for bats calculated with the analytical model of spatial detection volume. Columns show the volume in km³ for four different clutter thresholds, each with and without a CSF. CSF is always 0.16 m higher than radar. Volume increase factor given in the grey boxes (radar image was analyzed between 25° - 120°, h_R_ = 2.04 m, h_CF_ = 2.2 m, d_CFR_ = 6 m, σ = 12,7 cm², P_min_ = -74 dBm).

Changing the CSF geometrical setup influences not only the shape of the maximum detection range curve in the 2D simulation diagram (Fig 9), but also the amount of land clutter on the radar image since both effects are cross-linked. The best optimization in terms of the largest radar detection volume for bats with the CSF utilized in this study (e.g. 6 m radius and 2.2 m in height) is dependent on the tolerable clutter threshold (definition: see input 6 in the analytical volume model) and of the height difference from the CSF to the radar antenna (h_CFR_). Results of the detection volume simulated with the analytical model for the utilized radar system, which also takes the experimental results as an input, are given in Fig 10. Four different clutter thresholds were considered, each with (orange column) and without a CSF (blue column). Independent of the clutter threshold, h_CFR_ was determined to be ideally 0.16 m as for lower and higher h_CFR_ the detection volume decreases.

The maximum relative detection volume increases to more than double the volume (factor 2.5, grey box in Fig 10), when using a CSF and a threshold set to a level of 0. Even with higher clutter thresholds, the volume increase is in all cases huge and constant close to factor 2, showing the high optimization achieved with the CSF. The absolute detection volume increases with higher clutter thresholds, as more regions or pixel (see Eq (11)) on the radar image reaching up to the threshold level will be considered in the model. For the optimized case, the absolute detection volume is more than five times bigger, when comparing a threshold level of 24 with a level of 0. Selection of an appropriate threshold level and determining the absolute volume is depending on the subsequent data processing methods. Manual and automatic processing must be capable of detecting a bat echo even in clutter up to the set threshold level [16, 17]. It can be argued that the clutter threshold could be set to a level below the mean intensity threshold for echo visibility of 25. This would allow objects still to be distinguishable from background clutter. However, visual observations do not allow distinguishing the RGB composition of such close threshold levels. Furthermore, the clutter is not static and fluctuates between several threshold levels. This also limits the perceptibility when automated comparisons of thresholds are performed, as performed by the track extraction software radR [17].

Based on the current research and the explained difficulties, a clutter threshold of level 8 is recommended for further studies. Hereby the optimized volume includes 0.0105 km³ of airspace for the analyzed radar image section between 25° and 120°. Extrapolating for 360° radar operation, the volume is 270 times (rounded) bigger compared to a single acoustic bat detector, assuming a hemisphere shaped microphone detection characteristic with radius of 40 m. Of course, this is an approximation, and is highly depended on the landscape parameters influencing the radar image. However, it very well shows the advantages of the pulse radar.

#### Recommendations for practice

**Fig 11:**
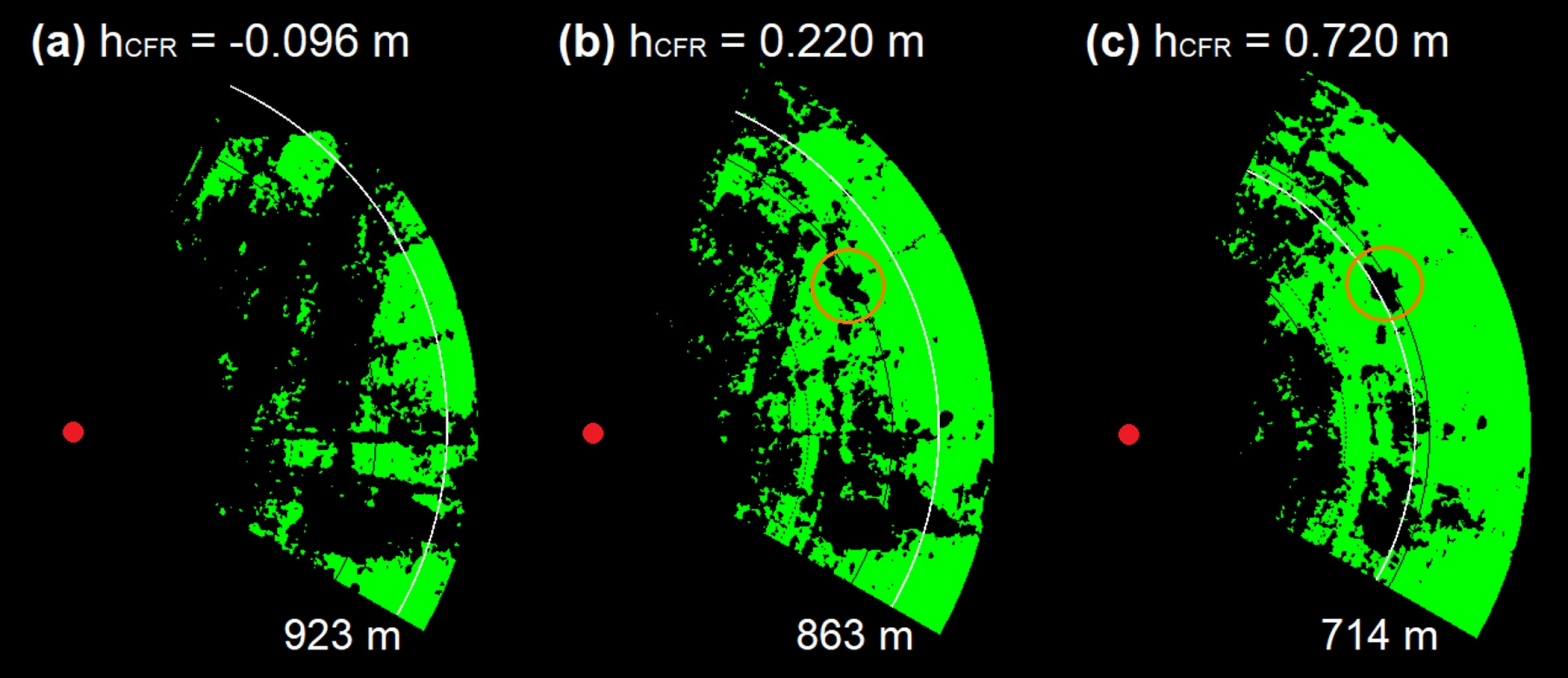
Variation in bat model visibility and maximum detection range of the radar shielded by a CSF section for three heights differences. Visible pixels are shown in green color (clutter threshold level of 8); the red dot shows the radar position; the white circle section shows the maximum detection range simulated for the bat model; the orange circle shows a visible turbine (h_CF_ = 2.2 m, d_CFR_ = 6 m, σ = 12,7 cm², P_min_ = -74 dBm).

A taller CSF (h_CFR_ > 0) results in better clutter reduction at ground level, because more ground directed radiation is shielded, improving the radar visibility. At the same time, the lower detection boundary increases in height. This is a result of the geometrical depended Fresnel diffraction. In addition, a larger h_CFR_, decreases the maximum detection range. Less intense radiation from the outer radar beam, extracted from the antenna diagram, is emitted above the CSF, whilst the main beam is shielded. To conclude, exceeding h_CFR_ above 0.16 m minimizes the detection volume. Fig 11 shows the visible pixels of three radar images for different h_CFR_ values for the utilized CSF, while the clutter threshold is set to a level of 8 as defined in the best optimization section. The increase of visible pixels from Fig 11A to 11c is very well recognizable and shows the optimization of the radar image due to the shielding of the CSF. For example, Fig 11A does not show a turbine because of the heavy overlay of clutter, while the turbine is visible in Fig 11B (orange circle) and even better visible in Fig 11C. However, without considering the results from the advanced 2D detection range simulation, the radar images may mislead the observer to set the CSF to very high values of h_CFR_. Considering the detection ranges (plotted as white lines) might lead to a different selection of h_CFR_, as the detection range decreases from Fig 11A to 11c, where the turbine is almost out of detection range.

Based on the current CSF setup, which doubles the detection volume, practical recommended values for h_CFR_ are in the range of 0.15–0.4 m for bat in wind parks. When observing a single turbine in close distance, a large h_CFR_ value can be recommended. This helps for stronger clutter reduction, while the simultaneously higher positioned lower detection boundary is negligible and comes mainly into effect at large distances. Besides, a large detection volume is less important for this scenario. For both cases, the actual CSF dimensions offer a good compromise between material, physical space required and the achievable optimization of the radar image.

## Conclusions

In this study, we have shown that standard marine pulse radar offers a great potential to investigate the behavior of bats in wind parks for the development of risk mitigation measures. We conclude that it is not necessary to technically modify the radar itself in order to receive reliable results. However, to compare radar studies, calibration and the consideration of landscape parameters is required. This has been neglected in most previous research studies. By introducing a new analytical spatial detection volume model for a low-cost standard marine pulse radar, we presented a tool delivering comparable detection boundaries in 3D space for bat-sized objects. This model is easily adoptable to different field studies. The calibration inputs of the volume model were practically examined with a bat model and a metal sphere and delivered results in line with other studies. Nevertheless, the calculated detection boundaries are limited by a fixed RCS threshold, which does not automatically adopt to smaller RCS. The settings of the echo analysis procedure are currently chosen to be very conservative. By further modifying these settings, it is most likely, that the detection volume can be further improved.

To optimize the spatial detection volume, we have shown a clutter reduction method, utilizing a clutter shielding fence, which is necessary to track bats in an onshore environment. Simulation and experimental verification of the CSF characteristics incorporated in the analytic volume model have shown excellent compliance. We conclude that a CSF is a very helpful and comparatively cheap and an easily applicable tool for clutter reduction, without modifying the radar itself to improve the special detection volume. Nevertheless, even the optimized volume has still capabilities to be further increased. Therefore, further shielding techniques should be investigated, but also other methods of radar beam manipulation techniques could be suitable to improve the radar performance.

## Supporting information

S1 Fig Example radar image with large amount of clutter and an echo signature of a mean intensity level of 25 (3x3 pixel). (TIF)
