# Supplementary figures and images for: Analytical volume model for optimized spatial radar bat detection in onshore wind parks
Source: PLoS One. 2020 Sep 30;15(9):e0239911. doi: 10.1371/journal.pone.0239911 (PMC7526923; doi:10.1371/journal.pone.0239911)

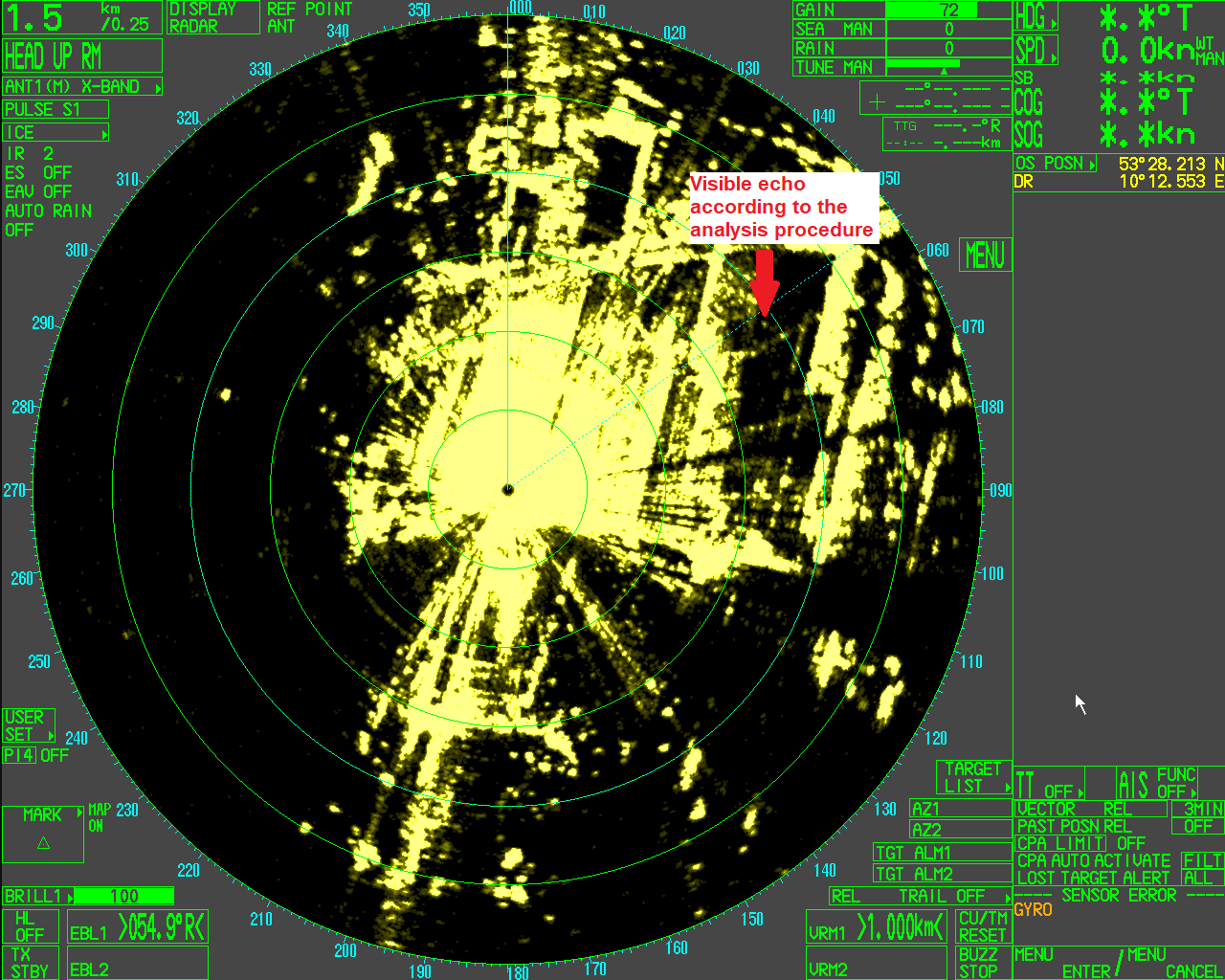

Supplement: S1 Fig — (TIF) [file pone.0239911.s001.tif]
